# A Blockchain-Based Trustworthy Model Evaluation Framework for Deep Learning and Its Application in Moving Object Segmentation

**DOI:** 10.3390/s23146492

**Published:** 2023-07-18

**Authors:** Rui Jiang, Jiatao Li, Weifeng Bu, Xiang Shen

**Affiliations:** College of Information Engineering, Shanghai Maritime University, Shanghai 201306, China; 202130310119@stu.shmtu.edu.cn (J.L.); 202010311045@stu.shmtu.edu.cn (W.B.); 202240310002@stu.shmtu.edu (X.S.)

**Keywords:** blockchain, deep learning, model evaluation, computer vision, moving object segmentation

## Abstract

Model evaluation is critical in deep learning. However, the traditional model evaluation approach is susceptible to issues of untrustworthiness, including insecure data and model sharing, insecure model training, incorrect model evaluation, centralized model evaluation, and evaluation results that can be tampered easily. To minimize these untrustworthiness issues, this paper proposes a blockchain-based model evaluation framework. The framework consists of an access control layer, a storage layer, a model training layer, and a model evaluation layer. The access control layer facilitates secure resource sharing. To achieve fine-grained and flexible access control, an attribute-based access control model combining the idea of a role-based access control model is adopted. A smart contract is designed to manage the access control policies stored in the blockchain ledger. The storage layer ensures efficient and secure storage of resources. Resource files are stored in the IPFS, with the encrypted results of their index addresses recorded in the blockchain ledger. Another smart contract is designed to achieve decentralized and efficient management of resource records. The model training layer performs training on users’ servers, and, to ensure security, the training data must have records in the blockchain. The model evaluation layer utilizes the recorded data to evaluate the recorded models. A method in the smart contract of the storage layer is designed to enable evaluation, with scores automatically uploaded as a resource attribute. The proposed framework is applied to deep learning-based motion object segmentation, demonstrating its key functionalities. Furthermore, we validated the storage strategy adopted by the framework, and the trustworthiness of the framework is also analyzed.

## 1. Introduction

Deep models play a central role in the research and application of deep learning. The study of deep models involves aspects such as model design, model training, model evaluation, and model interpretability analysis. Among these, model evaluation is a crucial step for measuring model performance and plays an important guiding and driving role in deep model research. Therefore, trustworthiness is a fundamental requirement for model evaluation. However, the current ways of evaluating deep models suffer from issues of untrustworthiness. Specifically, the complete process of model evaluation involves training, sharing, and evaluating. In the training phase, both the training data and training process are susceptible to attacks. When the training data comes from public datasets, there is a risk of direct tampering or backdoor attacks on the training data. Additionally, attackers can manipulate parameters and intermediate results to attack the training process. In the sharing phase, models contain sensitive information and intellectual property, making them potential targets for attacks. If models can be easily obtained, attackers can launch backdoor attacks on the models or adversarial attacks on the model inputs. In the evaluation phase, the issue of untrustworthiness arises from the current centralized evaluation method, leading to possibly incorrect or tamperable evaluation results, as well as potential security problems regarding data and models. There are two perspectives to address the aforementioned issues of untrustworthiness. One is to enhance the robustness of models against various types of attacks at the algorithmic level. The other is to build a well-designed model evaluation framework, based on a secure platform, to minimize the possibility of attacks and ensure the security and trustworthiness of data, models, and evaluation results. This paper focuses on the second perspective.

Blockchain is a widely applied distributed database based on cryptography, with advantages such as immutability and decentralization [1,2]. Smart contracts are self-executing codes or protocols deployed on the blockchain [3]. Access control technology is one of the core techniques in information security. Implementing access control in smart contracts can enable flexible and decentralized permission management for on-chain data [4,5,6,7,8,9]. Given the characteristics of blockchain and its related technologies, using them to address challenges in deep learning applications has become a research focus in recent years [10,11,12,13,14,15,16]. Inspired by these works and aiming to address the untrustworthiness issues in traditional deep model evaluation, this paper proposes to construct a blockchain-based model evaluation framework for deep learning, which can serve as a trustworthy platform for resource (i.e., data and models) sharing and model evaluation. The specific contributions are as follows.

(1) The proposed framework consists of an access control layer, a storage layer, a model training layer, and a model evaluation layer. The access control layer implements secure resource sharing using access control policies. To achieve fine-grained and flexible access control, the attribute-based access control model is adopted as the access control model, and user permissions are packaged as user role attributes using the principles of role-based access control. To decentralize and dynamically manage the access control model, a smart contract, Policy Contract, is designed to implement the above model, and the access control policies are stored in the blockchain ledger. The storage layer enables efficient and secure storage of resources. Resource files are stored using off-chain auxiliary storage based on the InterPlanetary File System (IPFS), and the encrypted results of index addresses of the resource files in the IPFS are stored in the blockchain ledger. To decentralize and efficiently manage the on-chain records of resources, a smart contract, Data and model record Contract (DC), is designed. The model training layer performs model training on the users’ server, and to ensure security, the training data must come from the storage layer. The model evaluation layer uses the data from the storage layer to evaluate the models stored in the storage layer. To achieve decentralized evaluation and ensure scores are immutable, a method is designed in DC to perform model evaluation, and the scores are automatically uploaded to the blockchain as a resource attribute.
(2) In the experimental section, the proposed framework is applied to the research of deep learning-based Motion Object Segmentation (MOS). Based on the aforementioned design, a blockchain-based deep model evaluation framework is constructed, which can be used for training, sharing, and evaluating deep MOS models. The experiment is set up with two users, the CDnet2014 dataset, and the FgSegNet_v2 model, demonstrating the key functionalities of the proposed framework. In the analysis part, the effectiveness of the framework’s storage strategy is validated, and the trustworthiness of the framework is also discussed.

The rest of this paper is organized as follows. Related works, including deep learning model research and its trustworthiness issues, deep model research for MOS and its trustworthiness issues, blockchain techonology and its applications in deep learning, are introduced in Section 2. The proposed framework is described in Section 3. The application of the proposed framework in MOS and related analyses are presented in Section 4. We conclude this work in Section 5.

## 2. Related Works

### 2.1. Deep Learning Model Research and Its Trustworthiness Issues

Deep Learning (DL) [17] is a type of machine learning method. It originated from research on neural networks and emerged in the early years of this century. The main idea behind DL is to learn the feature representation of data by constructing multi-layer neural networks and use these features for prediction and decision-making tasks. During the past two decades, with the development of Graphics Processing Units (GPU), artificial intelligence-specific chips, and computational frameworks, as well as the availability of large-scale datasets, research, and applications of DL have achieved significant progress [18]. The core of DL is deep models. In the computer vision field, the rise of Convolutional Neural Networks (CNN) was marked by AlexNet [19] winning the ImageNet [20] challenge, and since then, classical CNN models, such as the Visual Geometry Group (VGG) network [21], GoogLeNet [22], and Residual Network (ResNet) [23], have emerged, followed by CNN-based models for detection and segmentation [24]. In the natural language processing field, deep models, such as Recurrent Neural Networks (RNN) [25], Long Short-Term Memory networks (LSTM) [26], and Transformers [27], have been proposed for processing sequence data. In the field of game artificial intelligence, AlphaGo [28], developed by DeepMind, is a milestone in the development of deep reinforcement learning. In addition, there are deep models for data generation, such as AutoEncoders (AE) [29,30] and Generative Adversarial Networks (GAN) [31], as well as Graph Neural Networks (GNN) [32] for graph data. The main stages of deep model research include model design, model training, model evaluation, and model interpretability analysis, etc. Model evaluation involves training, sharing, and evaluation steps, and their trustworthiness and security issues have been actively investigated recently.

In terms of model training, DL relies not only on the quantity but also on the quality of data. If training data have been attacked, the reliability of the trained model will be compromised. Currently, there are several ways to attack training data: directly adding, deleting, or corrupting data [33], which may lead to overfitting, underfitting, and other problems, thus affecting the model’s generalization ability; backdoor attacks during training [33,34], i.e., injecting malicious samples into the training data to implant backdoors into the model, leading to incorrect predictions for specific inputs. This requires ensuring the integrity, security, and confidentiality of the training data. In addition to being affected by the training data, DL can also be influenced by the execution of the training [33,35], which is also susceptible to attacks. For example, attackers can tamper with the training parameters, such as the learning rate and the optimization algorithm, or tamper with intermediate results of the training, such as gradients. This requires that the model training process should be carried out on a secure platform, and the entire process can be monitored and traced.

In terms of model sharing, models involve sensitive information and intellectual property, and may become targets for stealing and attacking. Attackers can launch various attacks by attacking the model, such as directly destroying the architecture and parameters of the model, or more covert backdoor attacks against the model [36], as well as adversarial attacks [33,37]. Backdoor attacks on models refer to that attackers directly, adding backdoor components to the model, causing the model to produce incorrect outputs when specific backdoor trigger conditions are met. Adversarial attacks, on the other hand, refer to attackers studying the model and adding carefully designed interferences to the model inputs (i.e., data or their pre-processed results), which may be difficult to distinguish by the naked eye, leading the model to produce incorrect output results. Adversarial attacks can be divided into two types: white-box attacks and black-box attacks [38]. In white-box attacks, attackers have complete access to the internal structure and parameters of the model, allowing them to design sophisticated adversarial samples to attack the model. In black-box attacks, attackers can only access the inputs and outputs of the model and cannot directly access its internal structure and parameters. Therefore, they need to repeatedly query the model’s output to design adversarial samples. As can be seen, the premise of model sharing is the security, and model leakage can bring enormous risks to applications based on DL.

The trustworthiness of model evaluation involves the correctness, immutability, and diversity of the evaluation results, as well as the security of the evaluation process. Firstly, the input of evaluation is the model and the data, and the evaluation process is determined by the evaluation algorithm. The secure storage and sharing of these three determine the correctness of the evaluation results. Secondly, as a metric for measuring the model’s performance, the evaluation results should be immutable. Finally, model evaluation should ensure the diversity of the results. That is, a model should be evaluated multiple times by different users using different data and evaluation algorithms, then the resulting historical evaluation results can be used as credible evidence for the model’s performance. Traditional model evaluations are provided by model researchers, and the evaluation process mostly uses a few public datasets and evaluation algorithms, and the evaluation results are published by researchers in the academic literature. This makes it difficult to ensure the correctness, immutability, and diversity of the evaluation results. At the same time, the evaluation process cannot bring security risks to the model and the data.

In summary, providing a trustworthy model evaluation framework for DL research, ensuring the secure operation of critical steps, and preventing attacks on data, models, and evaluation results in related processes, is one of the key issues that need to be urgently addressed.

### 2.2. Deep Model Research for Motion Object Segmentation and Its Trustworthiness Issues

**Figure 1:**
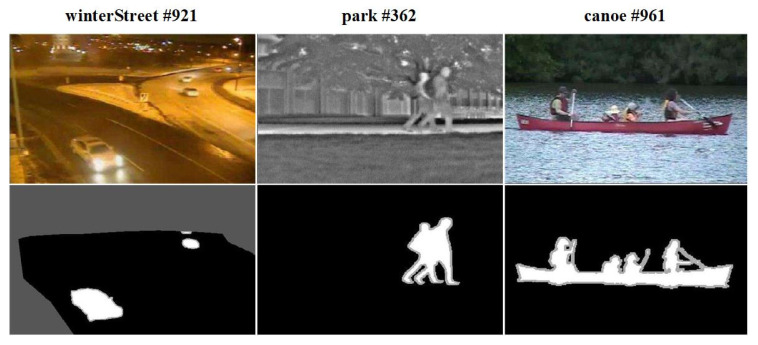
Three Video Frames (**top row**) and Their Ground Truth Masks (**bottom row**) From the CDnet2014 Dataset [41].

Motion Object Segmentation (MOS) [18,39,40] refers to the task of segmenting meaningful moving objects (also called foregrounds) in video frames, which is a critical step in video understanding and plays an important role in applications related to security, such as video surveillance and autonomous driving. In the video frame shown in the top row of Figure 1, moving vehicles, pedestrians, and canoes are all examples of moving objects that need to be segmented. With the development of DL and computer vision, DL-based MOS models have also been actively investigated. Among them, the mainstream methods are deep end-to-end models based on fully CNNs [18]. In the following, we will introduce the research status of deep MOS models from three aspects: training, sharing, and evaluation.

For the training of MOS models, training data consist of annotated video data. The best annotation is the pixel-level annotation, which means labeling each pixel in whole or part of each video frame as foreground or background, resulting in an image called a ground truth mask, as shown in the bottom row of Figure 1. The pixel-level annotation is fine-grained and usually requires manual completion, which incurs a large amount of manpower and time costs. Its difficulty in collection and its impact on model performance are both significant. In addition, there are other coarser annotations [42]. Currently, the datasets used in model research are mostly publicly available video datasets [43]. The important attributes of these video datasets related to MOS include Scene, Challenge, Sensor, Camera, Format, Annotation, etc., which are summarized in Table 1. After downloading the data, the model can be trained in a centralized manner on a local server or a third-party server, or it can be trained in a distributed manner by multiple clients. The attributes of deep MOS model training include training set, pre-training method, training method, and other training details, as shown in Table 2. The untrustworthy risks that may be encountered during training include: public datasets are attacked and the training data are tampered; third-party servers are attacked and the training data or the training process are tampered; a few clients used for distributed training are attacked and local training data or processes are tampered; some MOS model architectures are designed based on pre-trained models, and during training, all or part of the parameters obtained from pre-training are frozen. If these pre-trained models have been tampered with, it will also cause problems for the MOS model training.

In terms of model sharing, some methods have published their model files and training codes on Github, such as FgSegNets [44,45], BSUV [46,47], HOFAM [48], MUNet [49], GraphMOS [50], etc. The openness of Github allows anyone to access and use these models and codes. It provides functionalities, such as version control, code review, and issue tracking, allowing for collaborative development among multiple people. However, this openness also brings security issues. Attackers can easily obtain the model architecture and its training details, and develop attack strategies tailored to specific models and data. When an attack occurs, it may cause important moving objects to be ignored, resulting in incidents, such as the failure of a video surveillance system to monitor certain moving objects, or a traffic accident caused by the vision module of an autonomous driving system failing to see certain moving objects. Currently, a considerable number of DL-based MOS models and their training codes have not been published, which ensures security, but also limits the use and evaluation of these methods on a larger scale, thus constraining the development of DL-based MOS model research. Providing a secure and trustworthy MOS model-sharing platform has become one of the urgent problems to be solved.

In terms of model evaluation, the evaluation metrics for MOS models are quantitative standards used to measure the performance of the model. By inputting the model’s predicted masks and the ground truth masks into an evaluation algorithm, the model evaluation results under the evaluation metric can be outputted. Currently, frequently used model evaluation metrics for MOS are established based on the number of True Positives (TP), False Positives (FP), True Negatives (TN), and False Negatives (FN) of the predicted mask; the specific calculation formulas are summarized in Table 3 [51]. The evaluation setting refers to the method of selecting data for training and testing [39], which is another important aspect of DL-based MOS model evaluation. MOS model performance varies greatly under different evaluation settings. Currently, there are two types of evaluation settings: the Scene-Dependent Evaluation (SDE) and the Scene Independent Evaluation (SIE). SDE refers to using several labeled frames in a video as the training set and evaluating the model on frames from the same video. SIE refers to using labeled frames from several videos as the training set and evaluating the model on frames from completely different videos. Under the SDE setting, model performance is usually better than that under the SIE setting, but results under the SDE setting cannot evaluate the model’s robustness or cross-scene transferability, while results under the SIE can achieve that. An effective MOS model should be effective under different evaluation settings, and therefore, MOS model evaluation should adopt all of the above settings. Currently, MOS model evaluations are led by model proposers, thus centralized, especially when the model and related codes are not publicly available. This makes it difficult to ensure the correctness, immutability, and diversity of MOS model evaluation results, thereby limiting the development of DL-based MOS model research. Providing a secure, trustworthy, and decentralized MOS model evaluation platform is an effective way to change this situation.

### 2.3. Blockchain Technology

Blockchain is a distributed database based on cryptography in Point-to-Point network systems, proposed based on the Bitcoin protocol by Satoshi Nakamoto in 2018 [52]. Blockchain has the advantages of immutability, decentralization, etc., and has been widely used in fields such as data regulation, digital forensics, source tracing, and anti-counterfeiting [1,2]. Access control technology [53] is one of the core technologies of information security. These are the foundations for constructing the blockchain-based model evaluation framework for DL in this paper. Next, we will introduce related works from these aspects.

The immutability of blockchain is related to its structure. Blockchain links blocks together using hash algorithms, and the head of each block contains the hash value of the previous block. If the information in a block is changed, the hash value of the block is also changed, and it will no longer match the hash value stored in the next block, ensuring the immutability of the information in this block. Due to its immutability, Blockchain Technology (BT) is often used in fields such as system tracing and information certification [54,55,56,57]. The decentralization of BT includes two aspects. Firstly, the storage of blockchain is decentralized. Blockchain adopts a distributed ledger to record all transactions and state changes, so that each node has a complete copy of the ledger, avoiding problems such as untrustworthiness of third parties and single-point-failure. Secondly, there is no central organization or control system on the blockchain to manage and maintain the network. Each node can independently verify and update the ledger, and participate in the consensus process, thus ensuring the security and reliability of the data. The concept of smart contracts was proposed in the early 1990s [58]. Smart contracts have the characteristics of automation and programmability, and can encapsulate the complex behaviors of nodes in the blockchain. The decentralization of the blockchain ensures that smart contracts can be executed transparently and securely, and also makes the execution of smart contracts independent of any central authority or server. Smart contracts can also be used to flexibly embed various data and digital assets, thus achieving secure and decentralized on-chain information exchange and management [3].

In recent years, implementing access control on the blockchain through smart contracts has become a hot research topic [4,5,6,7,8,9]. Access control technology [53] is one of the core technologies of information security. It refers to assigning access permissions to a group of users so that they can obtain data, while restricting the ability, scope, or time for other users to access data and ensure that the information will not be accessed by unauthorized users. Common access control models include a Role-Based Access Control (RBAC) model [59] and Attribute-Based Access Control (ABAC) model [60]. RBAC is an access control method that implements enterprise security policies. By adding a set of roles, it reduces the complexity of directly assigning permissions to users. In situations where there are a large number of users or system permissions, it can greatly reduce the time spent on repetitive operations and improve system management efficiency. ABAC is a fine-grained access control model, and its main advantages are flexibility and scalability. It determines whether to grant access to the requester by judging whether the request contains the correct attributes, thus better addressing complex security requirements. Attributes are the core of ABAC, and basic attributes consist of the attributes of the access subject, the attributes of the object being accessed, and the attributes of the environment during access control permission verification. Each attribute instantiation constitutes an access control instance. Based on the different specifications of the subject, access control instances can be divided into access control policies and access control requests. Access control policies are typically defined in advance by resource owners or regulatory authorities to specify the access control rules for resources. Access control requests are submitted by resource visitors during access, and need to be compared with the pre-defined access control policies, which is referred to as access control permission verification. Traditional centralized access control systems have the following problems: centralized storage of access control policies inevitably leads to single-point-failure, and the system requires third-party supervision and control, which exposes the risk of policy tampering by untrustworthy third parties. Implementing the access control model on the blockchain can effectively solve the above problems.

### 2.4. Blockchain for Deep Learning

Recent studies have shown that blockchain technology has been applied in research related to DL [61]. For example, Hannah et al. [10] proposed a data collection scheme for the IOT based on DL and blockchain, using blockchain to provide secure data to DL, and identifying disease types using DL based on the collected data; Abraham et al. [11] proposed using DL to predict the occurrence of diseases based on pharmacogenomics data, and a blockchain-based platform was used to share medical data and model prediction results; Kuo et al. [12] proposed a consensus algorithm that emphasizes the importance of sharing deep models among peers in a blockchain; Rohani et al. [13] used BT to efficiently and securely share data and deep models in vehicular networks to estimate positioning errors; Goel et al. [14] used blockchain technology for secure storage of CNN models, proposing to store each layer of the model in a block of the blockchain, using the blockchain’s hash chain structure to provide tamper resistance and security verification for the model; Wang et al. [15] used BT to build a trusted machine learning analysis framework, which automates machine learning through smart contracts and stores models in a special binary format on the blockchain. Ref. [15] also applied this framework to computer vision and discussed how to enhance its data efficiency to meet the needs of edge computing. To solve the privacy and auditing issues of traditional distributed machine learning, Weng et al. [16] used BT to implement distributed machine learning, providing data confidentiality, computational auditability, and incentives for all parties to collaborate.However, we have not found any framework-level work that applies BT to model evaluations for DL. Furthermore, in terms of technical details, the strategies adopted in the proposed framework differ from existing works. Specifically, regarding the storage of data and models, the strategy of storing data or models directly on the blockchain can lead to an increasing burden, affecting its scalability and throughput. To address this issue, the proposed framework adopts a combined approach of on-chain and off-chain storage. In terms of data and model sharing, the proposed framework introduces access control into the blockchain to manage the access permissions of data and models, which has not been seen in existing works.

## 3. Proposed Blockchain-Based Trustable Model Evaluation Framework for Deep Learning

**Figure 2:**
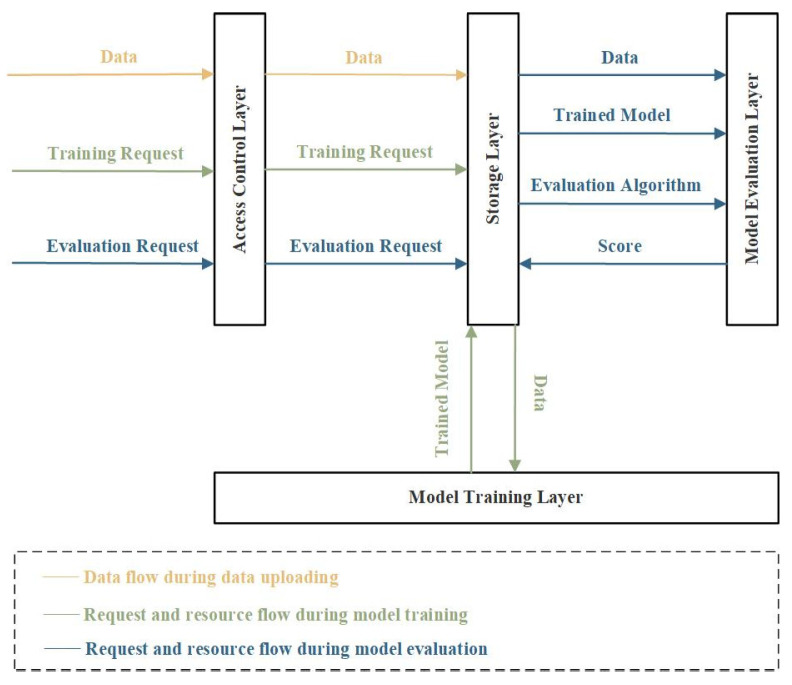
Proposed Blockchain-based Trustworthy Model Evaluation Framework for Deep Learning.

In order to address the untrustworthy issues involved in training, sharing, and evaluation during model evaluation in current DL research, a blockchain-based trustworthy model evaluation framework for DL is proposed. The framework consists of an access control layer, a storage layer, a model training layer, and a model evaluation layer, as shown in Figure 2. In the following, each layer will be described in detail. The functions used in algorithms can be found in Table 4. Hereafter, we refer to the data and models as resources, which are distinguished when necessary, and refer to the model performance evaluation results as scores.

### 3.1. Access Control Layer

For securely sharing resources, the proposed framework utilizes access control policies to manage on-chain operations. To achieve fine-grained and flexible access control and reduce the additional overhead caused by redundant operations, the ABAC model is adopted as the access control model. The idea of an RBAC model is utilized to bundle user permissions into roles as one of the user attributes in ABAC. The specific design of the access control model is as follows. An access control policy is defined as Policy={AS⋃AO⋃AE}. AS represents the attributes of a user (i.e., a subject), AS={UserID,Role}, where UserID is the unique identifier for the user, and Role represents the user’s identity, determined by the user’s permission for a specific resource, and can be Owner or Visitor. Users with the Owner role have permission to access, download, and save the resource, and can also add or revoke the Visitor role for other users. Users with the Visitor role only have permission to access and download this resource, and can not save it. Users without any role have no permission for this resource. AO represents the attributes of a resource (i.e., an object), AO={ResourceID,Type}, where ResourceID is the unique identifier of the resource in the blockchain, and Type represents its type, i.e., Data or Model. AE represents the attributes of the environment. In this framework, to achieve time-based access control, the environment attribute is set to the system time when the policy is submitted.

The structure of each access control policy recorded in the blockchain ledger is set as {PolicyID,Policy}. PolicyID is the unique index of the policy, and calculated as PolicyID=SHA256{AS.UserID+AO.ResourceID+AS.Role}, where the SHA256 is a frequently used cryptographic hash function of the SHA-2 hash family [62] and has properties such as collision resistance and one-wayness. Users’ operations on the blockchain must go through the access control verification with steps as follows. Firstly, an access control request for an operation is generated as ACRequest={UserID,ResourceID,Role,OP}, where OP represents the user’s operation on the resource. Then, an index of the request is generated by ACRequestID=SHA256{ACRequest.UserID+ACRequest.ResourceID+ACRequest.Role}. With the ACRequestID, we can try to retrieve an access control policy from the blockchain state database. If no policy is found, it means that the user has no permission for this operation. If a policy is found, a further check is needed to verify if the user’s role for the resource satisfies the permission for this operation.

In order to achieve decentralization and dynamic management of the access control model, a smart contract, called Policy Contract (PC), is designed. The main methods of PC are as follows. AddPolicy() adds an access control policy to the blockchain ledger and updates the blockchain state database. This method is used in the following two cases. One is to call this method to add the Owner role for a user when this user uploads a resource to the blockchain. The other is that the resource owner calls this method to add the Visitor role for other users. The implementation of AddPolicy() is shown in Algorithm 1. QueryPolicy() is used to search an access control policy in the blockchain state database using an index as the input. If a policy is found, the method returns the information of the policy. Otherwise, it will return an error message. DeletePolicy() deletes the access control policy with the input index from the blockchain state database. CheckAccess() generates an access control request for the user’s current operation and verifies whether the user’s permission for this operation is valid or not, returning True or False.

**Algorithm** **1** AddPolicy(): Adding an access control policy to the blockchain**Require:** AS,AO **Ensure:** OKorError 1: **if** AS==NullorAO==Null **then** 2:  **return** Error(“TheInputisnotvalid.”) 3: **end if** 4: PolicyID←SHA256(AS.UserID+AO.ResourceID+AS.Role) 5: Response←APIstub.GetState(PolicyID) 6: **if** Response!=Null**then** 7:  **return** Error(“Thepolicyalreadyexists.”) 8: **end if** 9: AE.Time←Linux.time.Now() 10: Policy←Marshal(AS,AO,AE) 11: APIstub.PutState(PolicyID,Policy)12: **return** OK

### 3.2. Storage Layer

**Figure 3:**
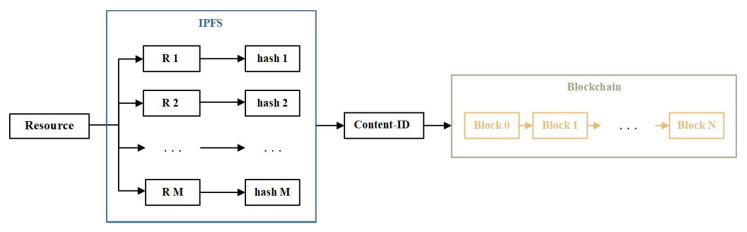
Storage Status of A Resource File in the Storage Layer (R *i*: a small chunk of the resource file stored in the IPFS; hash *i*: the hash value of R *i*; Block *j*: a block of the blockchain).

Resource files will occupy a large amount of storage space. With the increasing of nodes and transactions in the blockchain, the storage burden will gradually increase, resulting in difficulties for subsequent nodes to join the blockchain and limitations on the throughput rate. To alleviate the storage pressure on the blockchain and improve searching efficiency, an off-chain auxiliary storage method based on the InterPlanetary File System (IPFS) [63] is adopted. The IPFS is a content-addressed distributed file storage protocol with good security and high transmission speed. A file uploaded to the IPFS is split into small chunks, and these chunks and their hash values are stored on nodes across the network. The IPFS returns a unique hash value for each file, which is referred to as the Content-ID (CID) of this file in this paper. Users can download files from the IPFS from multiple servers simultaneously. Even if one node is temporarily offline, users can immediately switch to other nodes, avoiding resource loss and single-point-of-failure issues caused by network failures and other problems. Due to the content-addressable nature of the IPFS, the CID of each file is independent of server locations, the file name, etc. Furthermore, hash functions provide collision resistance, making it difficult for an attacker to steal a file from the IPFS as long as its CID has not been leaked. In the proposed framework, the resources are stored in the IPFS. To enable effective and secure resource sharing among the users, the CID of each resource is encrypted and stored in the blockchain ledger. The storage process of a single resource file from the IPFS to the blockchain is illustrated in Figure 3.

The structure of each resource record stored on the blockchain is set as Record={ResourceID,ResEncryAdr,ResInfo,EvalRecord}. Here, ResEncryAdr is the encrypted result of the CID of the resource in the IPFS by using the key of the uploader. ResourceID=SHA256(ResEncryAdr) is a message digest obtained by hashing the ciphertext with the SHA256 algorithm. Due to the strong collision resistance property of the SHA256, it is almost impossible to find or forge another data with the same hash result, so ResourceID can serve as the unique identifier for the resource record. ResInfo is the descriptive information about the resource, and EvalRecord is the historical evaluation records related to the resource with scores evaluated and uploaded by the model evaluation layer. An evaluation algorithm is used to evaluate the performance of the model and is a special resource. To ensure the immutability of evaluation algorithms, they are also stored in the IPFS and made available as public resources shared among users in the blockchain. Evaluation algorithms do not have the evaluation record attribute of data and models. Therefore, the storage structure of an evaluation algorithm record on the bockchain is set as EvalAlg={EvalAlgID,EvalAlgAdr,EvalAlgName}, where EvalAlgID represents the unique identifier of the evaluation algorithm record, EvalAlgAdr is the CID of the evaluation algorithm in the IPFS, and EvalAlgName denotes its name.

To achieve decentralized and efficient management of resource records in the blockchain, a smart constract, named Data and model record Contract (DC), is designed. The main methods of DC are as follows. AddResource() uploads a resource file to the IPFS and records its descriptive information and encrypted CID in the blockchain. This method is shown in Algorithm 2. FindByResID() is used to search for a resource record in the blockchain. ModelEvaluation() is used to evaluate the performance of a model and add the corresponding score to resource records. It is implemented in the model evaluation layer, as described in Section 3.4.

**Algorithm** **2** AddResource(): Uploading a resource to the storage layer**Require:** Resource,ResInfo,ResourceKey **Ensure:** OKorError 1: CID←UploadToIPFS(Resource) 2: **if** CID==Null **then** 3:  **return** Error(“AnerroroccurredwhileuploadingtheresourcetotheIPFS.”) 4: **end if** 5: ResEncryAdr←Encrypt(CID,ResourceKey) 6: ResourceID←SHA256(ResEncryAdr) 7: Response←APIstub.GetState(ResourceID) 8: **if** Response!=Null **then** 9:  **return** Error(“Theresourcerecordalreadyexists.”) 10: **end if** 11: APIstub.PutState(ResourceID,Marshal(ResEncryAdr,ResInfo))12: **return** OK

### 3.3. Model Training Layer

The model training layer is conducted on the users’ servers, and the training data must come from the data recorded in the blockchain to ensure data security. After the training is completed, the user can choose to upload the trained model and its related information to both the IPFS and the blockchain.

The operation process for a user to train a model is as follows. Firstly, the user submits a model training request, which is a request for accessing and downloading training data, i.e., TrainRequest=DataRequest, where DataRequest={UserID,DataID,DataKey,DataRole,OP}. Then, the smart contract PC of the access control layer determines whether the DataID has a corresponding record in the blockchain. If not, an error will be returned. If there is a record, the user’s permission for the data are verified based on the DataRequest. If the verification fails, an error will be returned. If the verification is passed, the CID of the requested data in the IPFS will be decrypted, and the user can obtain the data locally based on its CID. Finally, the user trains a model using the training data and obtains the trained model. If the user chooses to share the trained model, the model file will be uploaded to the IPFS, and a corresponding resource record will be added in the blockchain.

### 3.4. Model Evaluation Layer

The model evaluation layer uses data and an evaluation algorithm recorded in the blockchain to evaluate the performance of a model recorded in the blockchain, and uploads the score to the blockchain ledger. To achieve decentralization of the model evaluation process and immutability of the score, the entire evaluation process is conducted on the blockchain. When the evaluation is completed, the score will be automatically uploaded to the blockchain.

The operation process for a user to evaluate the performance of a model is as follows. Firstly, the user submits a model evaluation request, EvalRequest={ModelRequest,DataRequest,EvalAlgID}, where ModelRequest={UserID,ModelID,ModelKey,ModelRole,OP} and DataRequest={UserID,DataID,DataKey,DataRole,OP}. Then, the smart contract defined in the access control layer, PC, checks if there are corresponding records for ModelID, DataID, and EvalAlgID in the blockchain. If not, it returns an error. If there are records, the PC further verifies if the user has access permissions for the corresponding resources based on ModelRequest and DataRequest. If the user fails the access permission verification for any of the resources, an error will be returned. If all verifications are passed, the CID of the requested resources in the IPFS will be decrypted, and the user will retrieve the data, model, and evaluation algorithm based on their CID. The smart contract defined in the storage layer, DC, uses the evaluation algorithm to calculate the score of the model on the given data, and the score will be packaged as an EvalRecord and uploaded to the blockchain. The evaluation record is designed as one of the attributes of a resource, and the storage structure of an evaluation record is designed as: EvalRecord={DataID/ModelID,EvalAlg,Score,Time}, representing the ID of resources used in the evaluation process, the evaluation algorithm, the generated score, and the evaluation time. The used method ModelEvaluation() of the DC, is shown in Algorithm 3.

**Algorithm** **3** ModelEvaluation(): Evaluating model performance and uploading scores to the blockchain**Require:** ModelRequest,DataRequest,EvalAlgID **Ensure:** OKorError 1: AccessFlag=(CheckAccess(ModelRequest)&CheckAccess(DataRequest)) 2: **if** AccessFlag==False **then** 3:  **return** Error(“Noaccesspermission.”) 4: **end if** 5: /**Obtaining the models and data required for evaluation**/ 6: ModelRecord←APIstub.GetState(ModelRequest.ModelID) 7: ModelAdr←Decrypt(ModelRecord.ResEncryAdr,ModelRequest.ModelKey) 8: Model←DownloadFromIPFS(ModelAdr) 9: DataRecord←APIstub.GetState(DataRequest.DataID) 10: DataAdr←Decrypt(DataRecord.ResEncryAdr,DataRequest.DataKey) 11: Data←DownloadFromIPFS(DataAdr) 12: EvalAlgRecord←APIstub.GetState(EvalAlgID) 13: EvalAlg←DownloadFromIPFS(EvalAlgRecord.EvalAlgAdr) 14: /**Evaluating model performance and uploading scores**/ 15: Score←Eval(Model,Data,EvalAlg) 16: ModelScoreRecord←Marshal(DataRequest.DataID,EvalAlg,Score,Linux.time.Now()) 17: ModelRecord.EvalRecord.Add(ModelScoreRecord) 18: APIstub.PutState(ModelRequest.ModelID,Marshal(ModelRecord)) 19: DataScoreRecord←Marshal(ModelRequest.ModelID,EvalAlg,Score,Linux.time.Now()) 20: DataRecord.EvalRecord.Add(DataScoreRecord) 21: APIstub.PutState(DataRequest.DataID,Marshal(DataRecord))22: **return** OK

## 4. Experiment and Analysis

In this section, we design an experiment to apply the proposed model evaluation framework to DL-based motion object segmentation (MOS), demonstrating the functionalities of the proposed framework. Subsequently, we analyze the performance of the framework.

### 4.1. Experimental Settings

In this experiment, based on the design in Section 3, we built a blockchain-based Model Evaluation Framework (MEF), and used it to train, share, and evaluate deep MOS models. We used a PC to build a blockchain network environment based on the Hyperledger Fabric [64], and established a distributed container network, based on the Docker Swarm platform, to simulate distributed nodes. The experiment was set up with two users, User1 and User2, with User2 conducting deep model training using an Intel i9-10980XE CPU and an RTX 3090 GPU.

The dataset used is the CDnet2014 [41]. The CDnet2014 contains 53 videos of real-world scenes under 11 challenge categories, including: Dynamic Background, Camera Jitter, Shadow, Intermittent Object Motion, Thermal, Bad Weather, Low Frame-Rate, Night, Pan-Tilt-Zoom, Air Turbulence, and Baseline. Videos of CDnet2014 have different lengths and sizes, and are partly annotated, pixels in each ground truth mask are classified into five categories: the gray value of the uninteresting part is 85, pixels in the region of interest are divided into static pixels, moving pixels, shadow pixels, and unknown category pixels, and their gray values are marked as 0, 255, 50, 170, respectively. We selected the video “highway” and its annotations from the “Baseline” category, as well as the video “boats” and its annotations from the “Dynamic Background” category as the experimental data, and named these two datasets as “highway” and “boats”, respectively.

**Figure 4:**
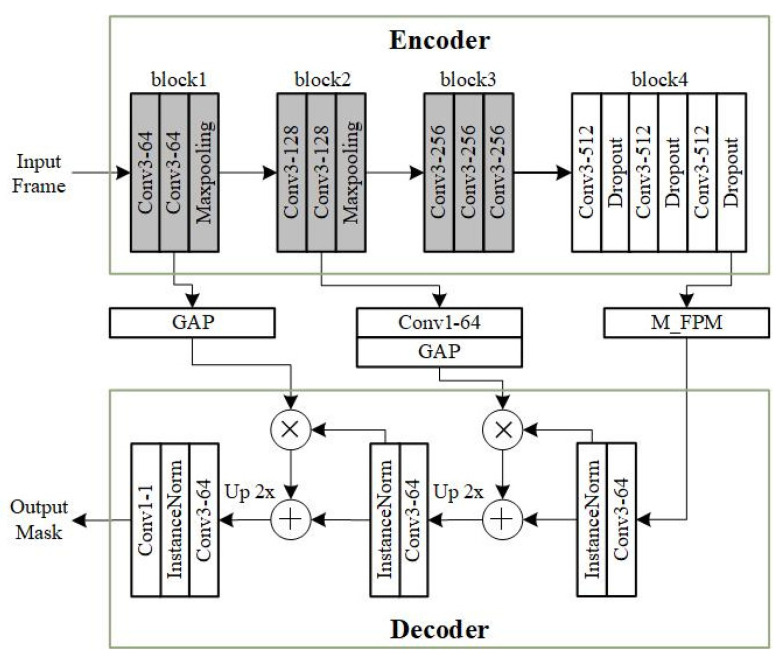
Architecture of FgSegNet_v2 [45] (Convi-j: j convolutions of i × i; GAP: global average pooling; M _ FPM: feature pooling module; InstanceNorm: instance normalization. Boxes filled with gray indicate layers that are frozen during training).

The deep MOS model employed is the classic foreground segmentation network FgSegNet_v2 [45,65]. FgSegNet_v2 is a single-input model. Its encoder comes from the first four blocks of the pre-trained VGG-16 [22], and max pooling layers after the third and fourth blocks are removed. To avoid over-fitting, dropout layers are added to the fourth block, and only the fourth block is set to be trainable. After the encoder, there is a Feature Pooling Module (FPM) that implements multi-scale feature encoding. The decoder of FgSegNet_v2 consists of three 3×3 convolutional layers, and a 1×1 convolutional layer, where each 3×3 convolutional layer is followed by an Instance Normalization layer. Two skip connections consisting of Global Average Pooling (GAP) are used between the encoder and the decoder, which help to fuse low-level features of the encoder and high-level features of the decoder, thereby reducing the loss of feature information and improving segmentation performance. The architecture of FgSegNet_v2 is shown in Figure 4. The model training was performed using Keras [66] with the TensorFlow backend. We followed the same training settings of [45]. Here, 80% of the training frames were used for training and 20% for validation. The batch size was set to 1. RMSProp [67] was selected as the training algorithm, and the specific parameters were set to ρ=0.9, $\epsilon =10^{-8}$. The initial learning rate was $10^{-4}$, and when the validation loss did not improve within five consecutive epochs, the learning rate was reduced by a factor of 10. The maximum epoch is 100. When the validation loss did not improve within ten consecutive epochs, the training stopped early. The value of each pixel output by the FgSegNet_v2 was between 0 and 1, and a threshold of 0.9 was used to binarize the network output to obtain the final predicted mask. The model evaluation algorithms used in the experiment are named after their corresponding model performance metrics, including Precision, Recall, and F-Measure, and their calculation methods are shown in Table 3.

### 4.2. Experimental Procedure

In this part, we introduce the key steps of this experiment and demonstrate the functionalities of the MEF.

**Figure 5:**
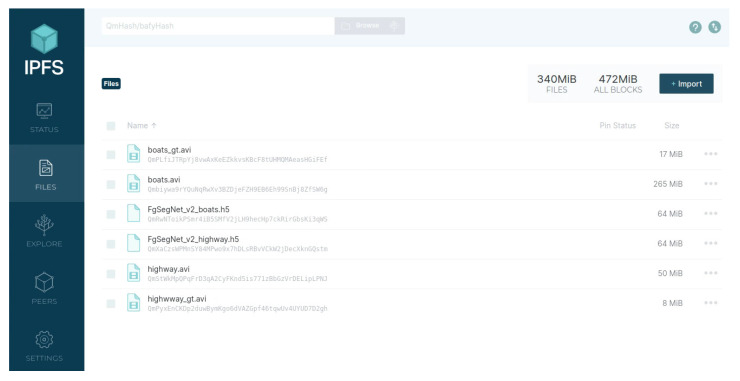
The IPFS-based Off-chain Storage of Data and Models in the Storage Layer of the MEF.

**Figure 6:**
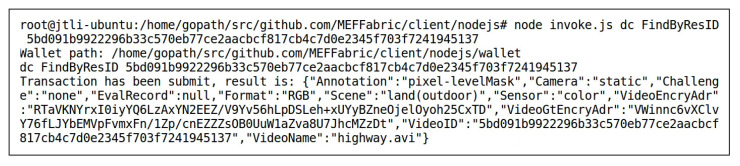
Invoking FindByResID() to Obtain the Record of the Data “highway” in the Blockchain After Uploading.

User1, the owner of the data “highway” and “boats”, uploaded the data to the storage layer of the MEF. Taking the data “highway” as an example, its storage status in the IPFS (https://ipfs.tech/ (accessed on 2 May 2023)) is shown in Figure 5, consisting of two files. The “highway.avi” is a video, and “highway_gt.avi” is the corresponding ground-truth masks. The string below each file name represents the CID, which serves as the index address of the file in the IPFS. The storage structure of the data “highway” in the blockchain is {VideoID,VideoEncryAdr,VideoGtEncryAdr,Re−sInfo,EvalRecord}, where VideoID is the unique identifier of the data “highway” recorded in the blockchain. VideoEncryAdr and VideoGtEncryAdr are the encrypted results of the CID of the video and its ground-truth mask in the IPFS, encrypted using an encryption key by User1, the ResInfo of the data “highway” is designed according to Table 1 as {VideoName:highway.avi,Scene:land(outdoor),Challenge:none,Sensor:color,Camera:static,Format:RGB,Annotation:pixel−levelMask}. Since the data “highway” had just been uploaded to the storage layer, there were no model evaluation records associated with it yet. Therefore, its evaluation record EvalRecord was null. Figure 6 shows the record of the data “highway” in the blockchain, which was obtained by invoking the FindByResID() method of the smart contract DC of the storage layer. The key-value pairs in the record were sorted alphabetically.

**Figure 7:**
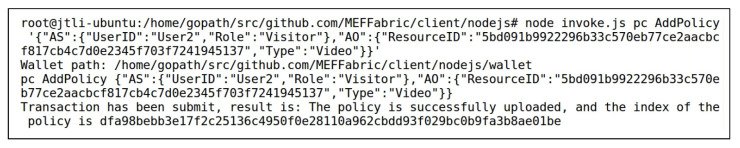
Invoking AddPolicy() to Grant User2 Access Control Permission for the Data “highway”.

**Figure 8:**
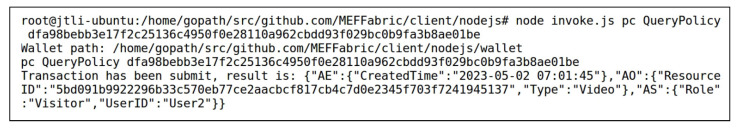
Invoking QueryPolicy() to Query the Added Access Control Policy Shown in Figure 7.

After the data “highway” and “boats” were stored in the storage layer of the MEF, User1 added access control permissions for User2 to access the data “highway” and “boats” by invoking the AddPolicy() method of the smart contract PC of the access control layer. Figure 7 shows User1 adding access control permission for User2 to access the data “highway”, with the ResourceID in Figure 7 matching the VideoID in Figure 6. Figure 8 demonstrates the QueryPolicy() method of the smart contract PC being used to verify the successful addition of the above access control policy. During the query, the index of the access control policy stored in the blockchain was used as the input after QueryPolicy(), as described in Section 3.1. The query result displays the complete access control policy added in Figure 7, indicating that the operation in Figure 7 was successful.

**Figure 9:**
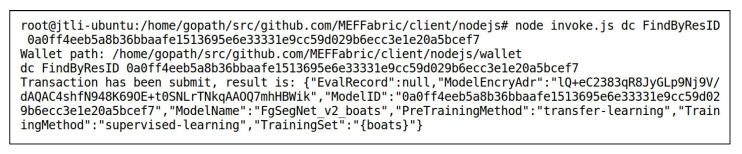
Invoking FindByResID() to Obtain the Record of the FgSegNet_v2_boats Model in the Blockchain.

**Figure 10:**
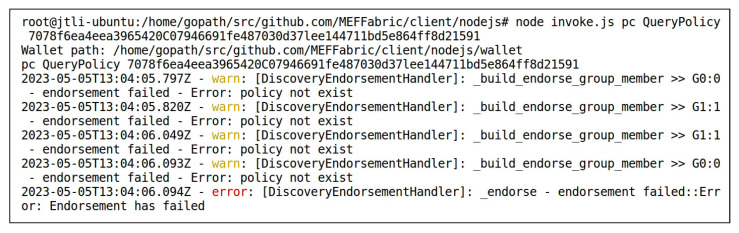
Invoking QueryPolicy() to Check if User1 Was Granted Permission to Access the FgSegNet_v2_boats Model.

User2 requested to train models using the data “highway” and “boats” separately. After passing the access control verification, User2 downloaded the data “highway” and “boats” to the local sever, and trained the FgSegNet_v2 model using the data “highway” and “boats” as training datasets, respectively, resulting in the models FgSegNet_v2_highway and FgSegNet_v2_boats. Finally, User2 uploaded these two trained models to the storage layer of the MEF. Taking FgSegNet_v2_boats as an example, its storage status in the IPFS can be seen in Figure 5, and its storage structure in the blockchain is {ModelID,ModelEncryAdr,

ResInfo,EvalRecord}, where ResInfo is designed according to Table 2 as {ModelName:FgSegNet_v2_boats,TrainingSet:{boats},PreTrainingMethod:transfer-learning,TrainingMethod:supervised−learning}. Since FgSegNet_v2_boats had just been uploaded to the storage layer, there were no model evaluation records associated with it yet. Therefore, its evaluation record EvalRecord was null. Figure 9 shows the record of the FgSegNet_v2_boats model in the blockchain, which was obtained by invoking the FindByResID() method of the smart contract DC. The key-value pairs in the record were sorted alphabetically. User2 granted User1 access permission to FgSegNet_v2_highway, while not granting access permission to FgSegNet_v2_boats. Therefore, when invoking the QueryPolicy() method of the smart contract PC to search for the corresponding access control policy, it shows that the policy did not exist, as shown in Figure 10. In Figure 10, the input following QueryPolicy() is the index of the access control policy stored in the blockchain, the calculation method of which is described in Section 3.1.

**Figure 11:**
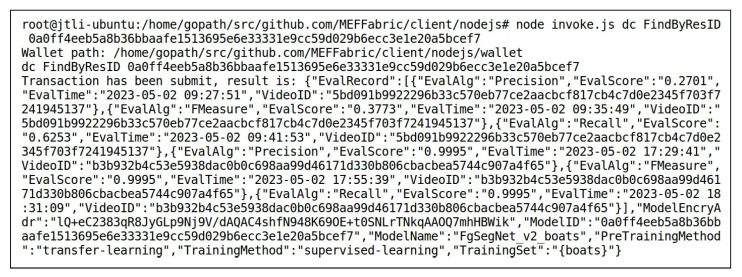
Invoking FindByResID() to Obtain the Record of the FgSegNet_v2_boats Model in the Blockchain After Model Evaluations.

**Figure 12:**
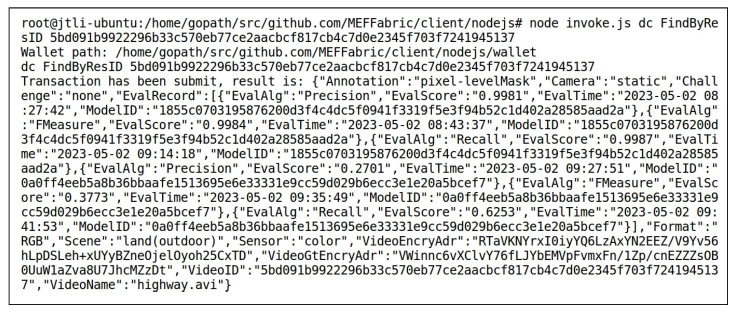
Invoking FindByResID() to Obtain the Record of the Data “highway” in the Blockchain After Model Evaluations.

User1 submitted a model evaluation request. After the access control verification was passed, User1 obtained the FgSegNet_v2_highway model, then evaluated the performance of the FgSegNet_v2_highway model using the data “highway” and “boats”, respectively, using Precision, Recall, and F-Measure as evaluation algorithms. This evaluation process generated six score records. Once the evaluation was complete, the score records were automatically uploaded to the blockchain. User2 also submitted a model evaluation request. After the access control verification was passed, User2 obtained the data “highway” and “boats”, then evaluated the performance of the FgSegNet_v2_boats model using the data “highway” and “boats”, resectively, using Precision, Recall, and F-Measure as evaluation algorithms. This evaluation process also generated six score records. Once the evaluation was complete, the score records were automatically uploaded to the blockchain. All the above evaluations and score recording processes were implemented by the ModelEvaluation() method of the smart contract DC. Figure 11 and Figure 12 respectively show the results of querying the records of the FgSegNet_v2_boats model and the data “highway” using the FindByResID() method of the smart contract DC after the model evaluation was completed. The retrieved key-value pairs were sorted alphabetically. It can be observed that the scores corresponding to the models and data were recorded in their EvalRecord attribute. The EvalRecord for the model is {VideoID,EvalAlg,EvalScore,EvalTime}, and the EvalRecord for the data is {ModelID,EvalAlg,EvalScore,EvalTime}. For the FgSegNet_v2_boats model, when the VideoID is “5bd0…5317”, the data used to evaluate the model is “highway”, and the evaluation setting is the SIE. Therefore, the scores generated by the three evaluation algorithms are not high, with values of 0.2701, 0.3773, and 0.6253, respectively. When the VideoID is “b3b9…4f65”, the data used to evaluate the model is “boats”, and the evaluation setting is the SDE. In this case, the scores generated by the three evaluation algorithms are relatively high, all of them being 0.9995. The evaluation under the SIE setting measures the model’s cross-scene transferability. The scores under different evaluation settings form a trustworthy model performance evaluation. For the data “highway”, when the ModelID is “1855…ad2a”, the model used for evaluation is FgSegNet_v2_highway, and the evaluation setting is the SDE. Therefore, the scores generated by the three evaluation algorithms are relatively high, with values of 0.9981, 0.9984, and 0.9987, respectively. When the ModelID is “0a0f…cef7”, the model used for evaluation is FgSegNet_v2_boats, and the evaluation setting is the SIE. In this case, the scores generated by the three evaluation algorithms are relatively low, consistent with the evaluation records of the FgSegNet_v2_boats model using the data “highway” mentioned above. The evaluation records of the video reflect the difficulty of conducting MOS for this video. If all models perform poorly in segmenting the video under the SIE setting, it indicates that there are challenges in conducting MOS on this video, and further research is needed.

### 4.3. Analysis

#### 4.3.1. Storage Strategy Analysis

**Figure 13:**
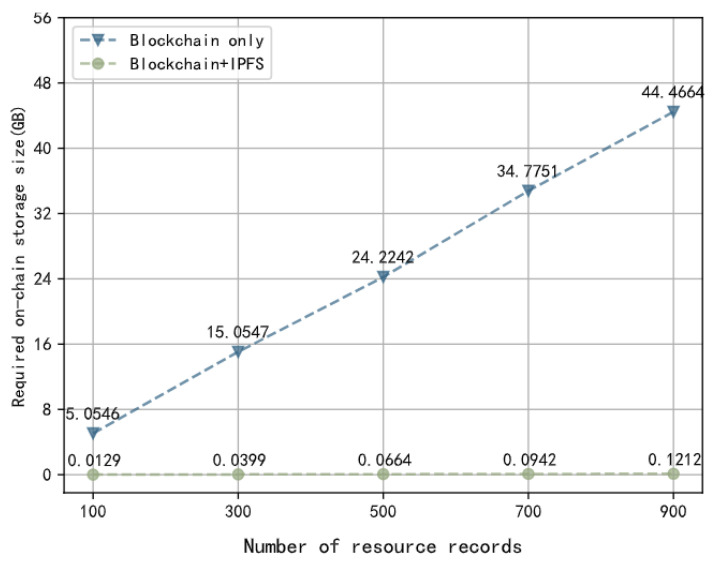
Performance Comparison between Two Storage Strategies.

A simulation experiment was conducted to validate the performance of the storage strategy adopted by the framework. The performance metric used is the required on-chain storage size. The comparison is made between the aopted storage strategy using a combination of blockchain and IPFS, referred to as “Blockchain+IPFS”, and the storage strategy using only blockchain, referred to as “Blockchain only”. The objects stored in the storage layer are resources. In practice, the size of resource files can vary and have no upper limit. To simplify the experiment, we assume that the size of resource files ranges from 1 MB to 200 MB. Based on statistics, each resource record requires 40 B to 240 B of on-chain storage space. We conducted five sets of experiments, where we measured the on-chain storage space required by “Blockchain+IPFS” and “Blockchain only” for different numbers of resource records: 100, 300, 500, 700, and 900. The results are shown in Figure 13. It can be observed that “Blockchain+IPFS” requires much less on-chain storage space compared to “Blockchain only”. As the number of records increases, the growth rate of on-chain storage space for “Blockchain+IPFS” is much slower than that of “Blockchain only”. This indicates that the adopted storage strategy combining blockchain and IPFS effectively reduces the storage burden on the blockchain.

#### 4.3.2. Trustworthiness Analysis

Based on the functionality demonstrated in Section 4.2, it can be observed that the proposed framework provides a certain level of trustworthiness in deep model evaluation. As analyzed in Section 2.1, the trustworthiness requirements for deep model evaluation include the security of resource sharing, the security of model training, the accuracy of model evaluation, the decentralization of model evaluation, and the immutability of model evaluation results. To ensure the security of resource sharing, the proposed framework incorporates IPFS as off-chain auxiliary storage, as shown in Figure 5. The address of each resource file in IPFS is encrypted and stored as an attribute in its resource record on the blockchain, as depicted in Figure 6 and Figure 9. Access control policies are also stored on the blockchain, and access control logics are deployed in a smart contract to manage access permissions for resources, as illustrated in Figure 7, Figure 8, and Figure 10. To ensure the security of model training, the framework requires that training data must have on-chain records. Similarly, for the correctness of model evaluation, the framework requires that both the evaluated model and data have records on the blockchain. To achieve decentralization in model evaluation and ensure the immutability of model evaluation results, the evaluation process is conducted by a smart contract. Upon completion of the evaluation, the evaluation results are automatically uploaded to the blockchain as one of the attributes of the resource, as shown in Figure 11 and Figure 12. Table 5 provides a comprehensive comparison of the trustworthiness between the proposed framework and the traditional deep model evaluation approach. The trustworthiness requirements of the deep model evaluation are displayed in the first column. Traditional approaches to deep model evaluation are detailed in Section 2.1 and Section 2.2, and the characteristics are summarized in the second column. The technical characteristics of the proposed deep model evaluation framework are summarized in the third column. By comparing them, the trustworthiness and innovation of the proposed framework can be observed.

## 5. Conclusions

In this paper, we propose a blockchain-based model evaluation framework for DL to minimize the issue of untrustworthiness in current model evaluation. The framework consists of an access control layer, a storage layer, a model training layer, and a model evaluation layer, and it has the following technical features:

(1) To achieve secure resource sharing, access control technology is introduced in the framework. The access control model adopts the ABAC model and incorporates the ideas of the RBAC model to enable fine-grained and flexible access permission division. A smart contract called PC is designed to manage access control policies in a decentralized and efficient manner.
(2) To achieve efficient and secure storage of resources, off-chain and on-chain storage approaches are combined. The IPFS is utilized for off-chain storage of resource files, while the encrypted results of their index addresses are stored in the blockchain. A smart contract called DC is designed to manage on-chain resource records in a decentralized and efficient manner.
(3) To ensure the security of training, in the model training layer, it is required that the training data must have records in the blockchain.
(4) To ensure the security of the evaluation process, the model evaluation layer must use the recorded data in the blockchain to evaluate the recorded models. A method in the smart contract DC is designed to enable decentralized evaluation and ensure the immutability of evaluation scores, which are automatically uploaded to the blockchain as a resource attribute.

While this framework addressed some untrustworthiness issues in the current deep model evaluation, it has not solved all the problems. Although the framework ensures the security of training data, the untrustworthiness issue in training still exists as the training process is conducted on users’ servers. Additionally, we assume that users use the framework on servers, but in practice, users are more likely to use mobile devices; this paper has not discussed how to use the framework on mobile devices. Furthermore, this framework is designed for deep model evaluation, and if it were to be extended to evaluate non-deep learning models, further work would be required. How to quantify the trustworthiness of the framework to support rigorous scientific validation also needs to be explored. All these issues need further research in future work.

## Figures and Tables

**Table 1:** Attributes of Video Datasets Related to MOS.

Attribute	Types
Scene	land (outdoor/indoor), water, underwater, etc.
Challenge	camera jitter, shadow, challenging weather, dynamic back- ground, intermittent object motion, low frame-rate, Pan-Tilt-Zoom camera (PTZ), night view, thermal imaging, air turbulence, occlusion, low-quality video, foreground size, etc.
Sensor	color sensor, thermal sensor, depth sensor, etc.
Camera	static camera, moving camera
Format	grayscale, RGB
Annotation	pixel-level mask, bounding box, etc.

**Table 2:** Attributes of Deep MOS Model Training.

Attribute	Types (or Descriptions)
Training Set	collection of names of training videos
Pre-training Method	transfer learning, self-supervised learning, etc.
Training Method	supervised learning, supervised adversarial learning, weakly supervised learning, etc.
Training Details	optimization algorithm, loss, batch size, maximum number of epochs, etc.

**Table 3:** Frequently Used Model Evaluation Metrics for MOS.

Metric (Abbr.)	Formula
Precision (Pr)	TP/(TP+FP)
Recall (Re)	TP/(TP+FN)
F-Measure	2×Pr×Re/(Pr+Re)
Specificity (Sp)	TN/(TN+FP)
False Positive Rate (FPR)	FP/(FP+TN)
False Negative Rate (FNR)	FN/(TN+FP))
Percentage of Wrong Classification (PWC)	100×(FN+FP)/(TP+TN+FP+FN)

**Table 4:** Descriptions of Functions.

Function	Description
APIstub.PutState(x,v)	Uploading *v* to the blockchain and setting its index as *x*
APIstub.GetState(x)	Searching with index *x* from the blockchain state database
Linux.time.Now()	Retrieving the current time in Linux system
Encrypt(x,k)	Encrypting *x* with key *k*
Decrypt(x,k)	Decrypting *x* with key *k*
UploadToIPFS(v)	Uploading *v* to the IPFS and return its CID
DownloadFromIPFS(x)	Downloading the file with CID *x* from the IPFS
$\mathrm{Marshal}(x_{1},x_{2},\ldots ,x_{n})$	Packaging $x_{1},x_{2},\ldots ,x_{n}$
Eval(m,d,e)	Using a dataset *d* and an evaluation algorithm *e* to evaluate the performance of a model *m*
Add(r)	Adding a record *r*

**Table 5:** Comparison of The Trustworthiness between The Proposed framework and The Traditional Deep Model Evaluation Approach.

Requirements	Traditional Deep Model Evaluation Approach	Proposed Framework
Security of Resource Sharing	Centralized storage of resources; resources are either completely private or completely public with no access control management.	Distributed storage of resources based on the IPFS; resource addresses are encrypted and recorded on the blockchain, and the management of resource records is governed by access control.
Security of Model Training	No explicit mechanism to ensure the security of training data.	The training data must be recorded on the blockchain.
Correctness of Model Evaluation	No explicit mechanism to ensure the security of the data and models used for evaluation.	The data and models used for evaluation must be recorded on the blockchain.
Decentralization of Model Evaluation	If the model is completely private, the model evaluation can only be performed by the owner of the model. If the model is completely public, the model evaluation can be done by any user.	The model evaluation requests are initiated by users who have access permissions to the corresponding resources, and are executed by a smart contract on the blockchain.
Immutability of Model Evaluation Results	The model evaluation results are recorded by the evaluator, and there is a risk of tampering.	The model evaluation results are automatically uploaded to the blockchain as a resource attribute.

## Data Availability

Publicly available datasets were used in this study. This data can be found here: http://changedetection.net/ (accessed on 1 May 2023).
